# The Neogene fossil record of *Aetomylaeus* (Elasmobranchii, Myliobatidae) from the southeastern Pacific

**DOI:** 10.1080/02724634.2019.1577251

**Published:** 2019-04-02

**Authors:** Jaime A. Villafaña, Giuseppe Marramà, Sebastian Hernandez, Jorge D. Carrillo-Briceño, Dirk Hovestadt, Rene Kindlimann, Jürgen Kriwet

**Affiliations:** 1 University of Vienna, Department of Paleontology, Althanstraße 14, Geocenter, 1090 Vienna, Austria, giuseppe.marrama@univie.ac.at; juergen.kriwet@univie.ac.at; 2 Biomolecular Laboratory, Center for International Programs, Universidad Veritas, 10105 San José, Costa Rica, shernandez@veritas.cr; 3 Sala de Colecciones Biológicas, Facultad de Ciencias del Mar, Universidad Católica del Norte, 1780000 Coquimbo, Chile; 4 Paläontologisches Institut und Museum der Universität Zürich, Karl Schmid-Strasse 4, CH-8006 Zürich, Switzerland, jorge.carrillo@pim.uzh.ch; 5 Merwedelaan 6, NL-4535ET Terneuzen, The Netherlands, dmhovest@zeelandnet.nl; 6 Zürichstrasse 58, 8607 Aathal-Seegräben, Switzerland, shark.collection@gmx.ch

## Abstract

The presence of eagle rays of the genus *Aetomylaeus* in the Neogene of the Temperate Pacific coast of South America (TPSA) still is ambiguous, although the fossil record of elasmobranch fishes (sharks, rays, and skates) from this area is quite good. Here, we present the first unmistakable fossil remains of *Aetomylaeus* from the Neogene of the TPSA. The material comprises 13 dental plates from one site in Peru and six localities in Chile ranging in age from Miocene to Pliocene and was compared with dental plates of extant species. Our study reveals that the number of tooth rows and the shape of lateral teeth in extant species are seemingly very variable and need to be established before fossil specimens can be confidently identified. Consequently, we do not assign the fossil specimens from the Neogene of the TPSA to any species but leave them as *Aetomylaeus*. Moreover, we recognized that only the shape of medial teeth provides reliable diagnostic characters in our material, whereas the shape and number of lateral teeth are highly variable, similar to the condition seen in extant species.

## INTRODUCTION

*Aetomylaeus* is one of the three extant eagle ray genera of the stingray family Myliobatidae sensu Naylor et al. (2012) and is sister to the genus *Myliobatis* (see also Marramà et al., 2018a). A fourth myliobatid genus, *Pteromylaeus*, introduced by Garman (1913), largely resembles *Aetomylaeus* but was differentiated from the latter by the presence of small, almost vestigial stinging tail spines. This character, however, is deemed insufficient for generic separation, and we follow Aschliman (2014), White (2014), Last et al. (2016), and the ‘Chondrichthyan tree of life project’ (https://sharksrays.org/) in considering *Pteromylaeus* to be a junior synonym of *Aetomylaeus*. This genus includes seven extant species that are distributed in the western Indian Ocean, Indo-West Pacific, and eastern central Pacific (Last et al., 2016; White et al., 2016). In the eastern central Pacific, only a single species, the mottled eagle ray *Aetomylaeus asperrimus*, has been reported off Panama and the Galapagos islands (Last et al., 2016).

The extant species occur mainly in tropical zones, but their specific habitat depends on their geographic distribution (Last et al., 2016). For instance, the roughskin eagle ray (*A. asperrimus)* from the eastern central Pacific is demersal on soft bottoms, whereas the duckbill eagle ray (*A. caeruleofasciatus*) from the eastern Indian Ocean is pelagic in coastal and inner continental shelves (Last et al., 2016; White et al., 2016). The depth range is not widely known in all seven extant species, but *A. bovinus*, *A. caeruleofasciatus*, and *A. vespertilio* occur from surface waters to depths of at least 100  m (Brito, 1991; Compagno, 1997; Last et al., 2016; White et al., 2016), whereas *A. asperrimus* and *A. maculatus* seemingly do not occur below 60  m depth (Myers, 1999; Love et al., 2005). *Aetomylaeus* feeds on crabs, hermit crabs, gastropods, bivalves, squids, prawns, worms, and bony fishes (Michael, 1993; Last et al., 2016). Ovoviparous reproduction (i.e., aplacental viviparity) was reported for *A. asperrimus*, *A. bovinus*, *A. maculatus*, and *A. milvus* (Dulvy and Reynolds, 1997; White, 2014).

The fossil record of myliobatids extends back into the Late Cretaceous; numerous extinct genera were erected for isolated teeth and/or dental plates from Cenozoic strata (Claeson et al., 2010; Adnet et al., 2012; Cappetta, 2012), and only two Paleogene taxa (*Weissobatis micklichi* and *Promyliobatis gazolai*) are known by complete and articulated skeletons (Hovestadt and Hovestadt-Euler, 1999; Marramà et al., 2018b). Unfortunately, the identities and numbers of fossil species assigned to *Myliobatis* and *Aetomylaeus* are confusing. Dental remains assigned to *Myliobatis* are very common in the fossil record, with the oldest dental remains coming from the Maastrichtian of Mali, and remains of this genus become very abundant in late Paleogene and Neogene strata (Claeson et al., 2010). Isolated teeth and dental plates assigned to *Pteromylaeus* also have been occasionally reported from Miocene and Pliocene sites in Europe (Cappetta, 2012) and tropical America (Carrillo-Briceño et al., 2018). *Aetomylaeus* is most common in the Neogene, and a few records occur in the late Eocene. However, the presence of earlier records is very ambiguous due to difficulties in identifying fossil myliobatid teeth (Hovestadt and Hovestadt-Euler, 2013; Engelbrecht et al., 2018). Eocene occurrences of *Aetomylaeus* have been reported from Hungary, the United Kingdom, U.S.A., and Uzbekistan (Hantken, 1875; Blake, 1941; Kemp, 1985; Case et al., 1996; Cicimurri and Ebersole, 2015), whereas Von Meyer (1844), Issel (1877), and White (1934) reported Oligocene records from Germany, Italy, and Nigeria. In the Neogene, several occurrences have been reported from Miocene sediments of Angola, Austria, Pakistan, and Portugal (Antunes, 1977; Hiden, 1995; Welcomme et al., 1997; Balbino and Antunes, 2006). Pliocene records are known from Libya and Spain (Mañé et al., 1988; Pawellek et al., 2012). In America, isolated teeth and dental plates of *Aetomylaeus* have been described from Miocene deposits of Brazil, Colombia, Cuba, Mexico, U.S.A., and Venezuela (Iturralde-Vinent et al., 1998; Carrillo-Briceño et al., 2016, 2018; Aguilera et al., 2017; Weems et al., 2017).

Although the fossil record of elasmobranchs along the eastern Pacific coast of South America is quite rich (Muizon and Devries, 1985; Carrillo-Briceño et al., 2013; Staig et al., 2015), Neogene remains of eagle rays of the genus *Aetomylaeus* are poorly represented in this area and only Suárez et al. (2004) and Gutstein et al. (2008) have indicated their presence without, however, providing description or figures. This might be related to the fact that morphological studies about their teeth and dental plates are scarce and the identification of fossil remains is consequently rendered very difficult and ambiguous (Claeson et al., 2010; Hovestadt and Hovestadt-Euler, 2013). The aim of this study is to describe and illustrate the first unambiguous fossil remains of *Aetomylaeus* from the Neogene of the southeastern Pacific. Unfortunately, it is not possible to assign the fossil dental plates to any species due to the high variation of dental morphologies in extant eagle rays (i.e., *Aetobatus*, *Aetomylaeus*, and *Myliobatis*) that have been evaluated in detail to date. Taxonomic descriptions at the specific level based on published material without clear indication of diagnostic characters urgently need to be revised (Hovestadt and Hovestadt-Euler, 2013; Engelbrecht et al., 2018). However, a revision of eagle rays based on dental characters is beyond the scope of this paper.

## MATERIALS AND METHODS

**FIGURE 1.**
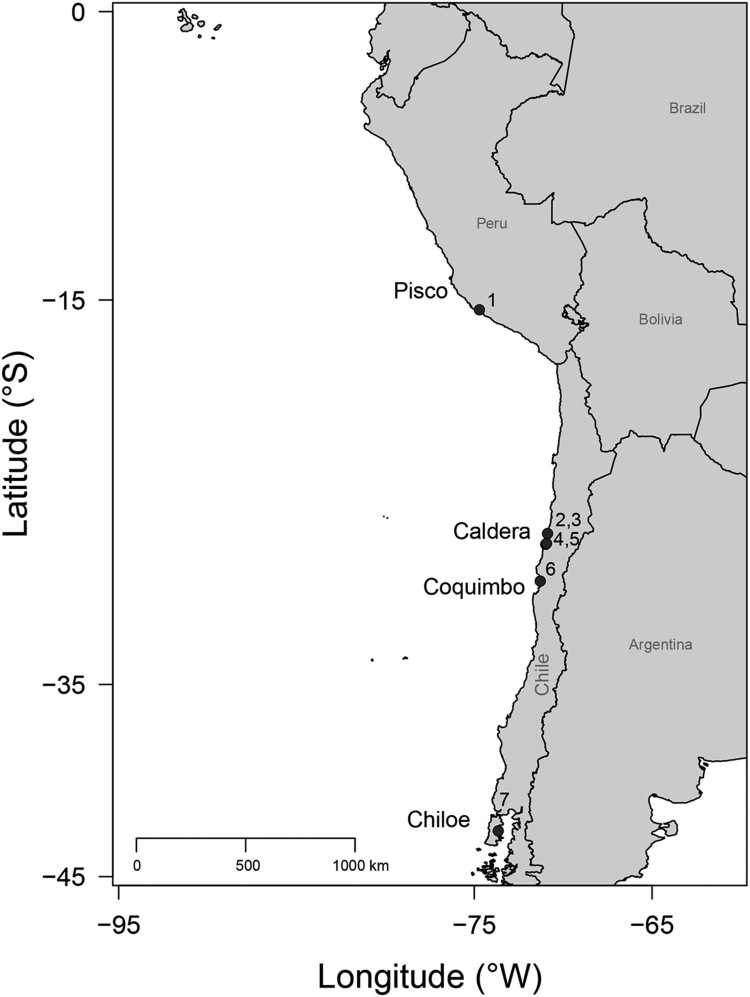
Location map of *Aetomylaeus*-bearing sites in the Temperate Pacific coast of South America (TPSA). **1**, Sacaco; **2**, Caldera; **3**, Mina Fosforita; **4**, Bahía Salado; **5**, Punta Chacos; **6**, Quebrada Honda; **7**, Lemuy.

The material that forms the focus of this study comprises 13 dental plates, which were recovered from different localities along the Temperate Pacific coast of South America (4°S to 42°S; TPSA hereafter), including one site in Peru (Sacaco) and six in Chile (Caldera, Bahia Salado, Punta Chacos, Mina Fosforita, Quebrada Honda, and Lemuy; Fig. 1).

**Institutional Abbreviations**—**CSIRO**, Commonwealth Scientific and Industrial Research Organisation, Hobart, Tasmania, Australia; **ERB**, Elasmobranch Research, F. Mollen, Berlaar, Belgium; **LACM**, Los Angeles County Museum of Natural History, Los Angeles, California, U.S.A.; **MCZ**, Harvard University Museum of Comparative Zoology, Cambridge, Massachusetts, U.S.A.; **MPC**, Museo Paleontologico de Caldera, Caldera, Chile; **MUSA**, Museo de Historia Natural e Historico de San Antonio, San Antonio, Chile; **NBC**, Naturalis Biodiversity Center, Leiden, The Netherlands; **NHMW**, Natural History Museum of Vienna, Austria; **RK**, R. Kindlimann private collection with public access, Uster, Zurich, Switzerland; **SCBUCN**, Sala de Colecciones Biologicas Universidad Catolica del Norte, Coquimbo, Chile; **UMMZ**, University of Michigan Museum of Zoology, Ann Arbor, Michigan, U.S.A.

**FIGURE 2.**
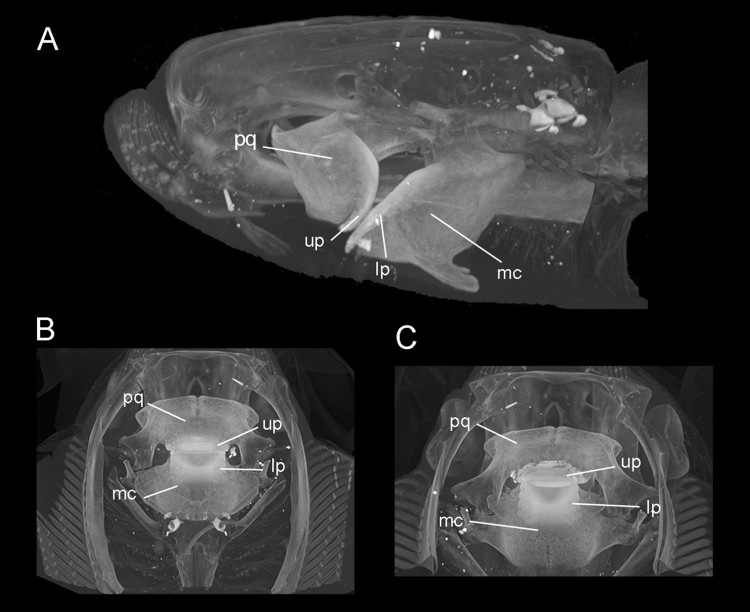
*Aetomylaeus nichofii* (MCZ S-1393; CT scans). **A**, lateral, **B**, dorsal, and **C**, ventral views. **Abbreviations**: **lp**, lower dental plate; **mq**, Meckel’s Cartilage; **pq**, palatoquadrate; **up**, upper dental plate.

**Comparative Material**—Upper and lower dental plates of *A. caeruleofasciatus* (CSIRO H 8109-01, adult male, 480 mm disc width) from the Gulf of Papua, Papua New Guinea; upper and lower dental plates of *A. nichofii* (LACM 38117.076, male, 519 mm disc width) from Pakistan; upper and lower plates of *A. nichofii* (MCZ S-1393, 250 mm disc width) from Indonesia; upper and lower dental plates of *A. bovinus* (NHMW 60727, adult female) from Italy; upper and lower dental plates of *A. bovinus* (ERB DUR02359.88, male) from Durban, South Africa. *A. milvus* (NBC 7465, male) from off Djakarta, Indonesia, and upper and lower dental plates of *A. maculatus* (UMMZ 191400) from Thailand were used for comparison. The position of the upper and lower plates within the jaws is shown in the extant species, *A. nichofii* (Fig. 2). The specimen MCZ S-1393 was computed tomographically (CT) scanned at the Harvard University Museum of Comparative Zoology.

**Terminology**—We follow the dental terminology used in Claeson et al. (2010), Cappetta (2012), and Hovestadt and Hovestadt-Euler (2013).

## GEOLOGICAL SETTINGS

The material that forms the focus of the present study comes from one locality in Peru (Sacaco) and six localities in Chile (Caldera, Bahia Salado, Punta Chacos, Mina Fosforita, Quebrada Honda, and Lemuy) (Fig. 1). The sediments of the northernmost locality, Sacaco (15°S, southern Peru), are part of the Pisco Formation (Muizon and Devries, 1985). This geological formation is characterized by the occurrence of mammals, sea birds, bony fishes, and chondrichthyans (Muizon and Devries, 1985; Marocco and Muizon, 1988; Stucchi, 2007). In particular, in the Sacaco area, five fossil sites are distinguished: El Jahuay, Aguada de Lomas, Montemar, Sud Sacaco, and Sacaco (Muizon and Devries, 1985). However, due to the lack of specific geographic coordinates of the material, we do not assign our material from Peru to a specific site in the Sacaco area. Based on strontium chemostratigraphic analysis (^87^Sr⁄^86^S), the Pisco Formation here ranges from 7.46 to 5.89 Ma (late Miocene; Ehret et al., 2012).

In northern Chile, the sediments of various fossil-bearing localities in the south of the Caldera region (27°S), such as Caldera, Punta Cachos, Bahia Salado, and Mina Fosforita, belong to the Bahia Inglesa Formation (Rojo, 1985; Marquardt, 1999). The Bahia Inglesa Formation includes some of the most important and richest fossiliferous sites in Chile (Suárez et al., 2004; Chavez, 2008; Pyenson et al., 2014), but its age still is controversial (Rojo, 1985; Marquardt, 1999; Achurra, 2004). Recently, Le Roux et al. (2016) dated each stratigraphic unit of the Bahia Inglesa Formation using ^87^Sr/^86^Sr isotopic information from marine mollusk shells. Accordingly, the basal-most unit has an age of 15.3 Ma (middle Miocene), whereas the uppermost one is dated at 2.4 Ma (lower Pleistocene).

The locality of Quebrada Honda is located in the north of La Serena (Coquimbo Region, Chile, 29°S). Neogene sediments in this region are part of the Coquimbo Formation, from which a diverse chondrichthyan fauna was reported (Staig et al., 2015). Studies based on the presence of marine invertebrates and dating of sedimentary units suggest an age ranging from the middle Miocene to Pliocene for the Coquimbo Formation (Le Roux et al., 2004, 2005).

The southernmost locality in Chile, Lemuy, is an island located in the Chiloé Archipelago (42°S). Deposits of this locality were assigned to the Lacui Formation (Sernageomin, 2004), and micropaleontological data suggest an early Miocene age (Nielsen and Glodny, 2009). Consequently, all material presented here comes from marine deposits in the TPSA ranging from early Miocene to early Pleistocene in age.

## SYSTEMATIC PALEONTOLOGY

Class CHONDRICHTHYES Huxley, 1880

Subclass ELASMOBRANCHII Bonaparte, 1838

Order MYLIOBATIFORMES Compagno, 1973

Family MYLIOBATIDAE Bonaparte, 1838 (sensu Naylor et al., 2012)

Genus *AETOMYLAEUS* Garman, 1908

*AETOMYLAEUS* sp.

(Figs. 3–7)

**FIGURE 3.**
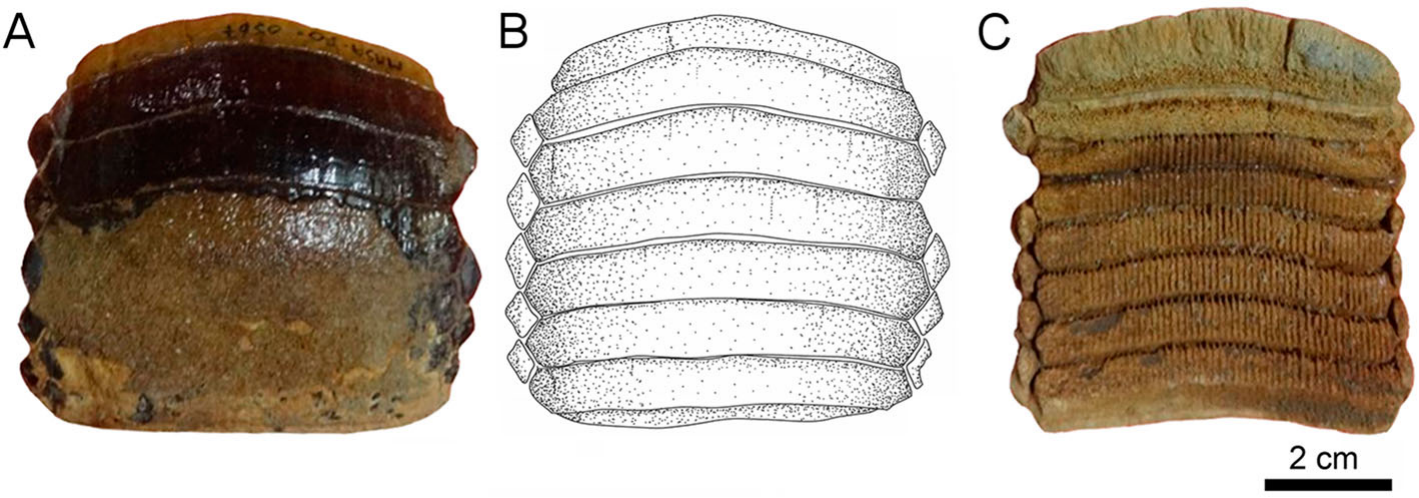
Fossil specimen MUSA-567 from the Bahia Inglesa Formation (middle Miocene–early Pleistocene), Bahia Salado, Caldera region, Chile. **A**, **C**, upper dental plate; **B**, reconstruction. **A, B**, occlusal and **C**, basal views.

**FIGURE 4.**
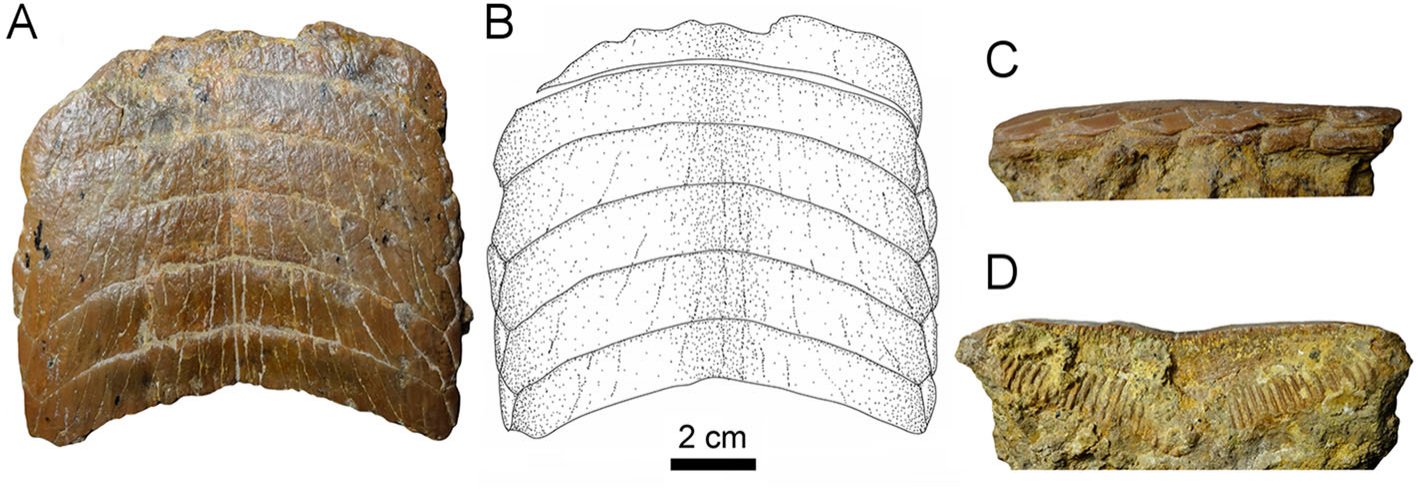
Fossil specimens RK/17-100 from the Bahia Inglesa Formation (middle Miocene–early Pleistocene), Caldera, Chile. **A**, **C**, and **D**, lower dental plate; **B**, reconstruction. **C**, lateral and **D**, lingual views.

**FIGURE 5.**
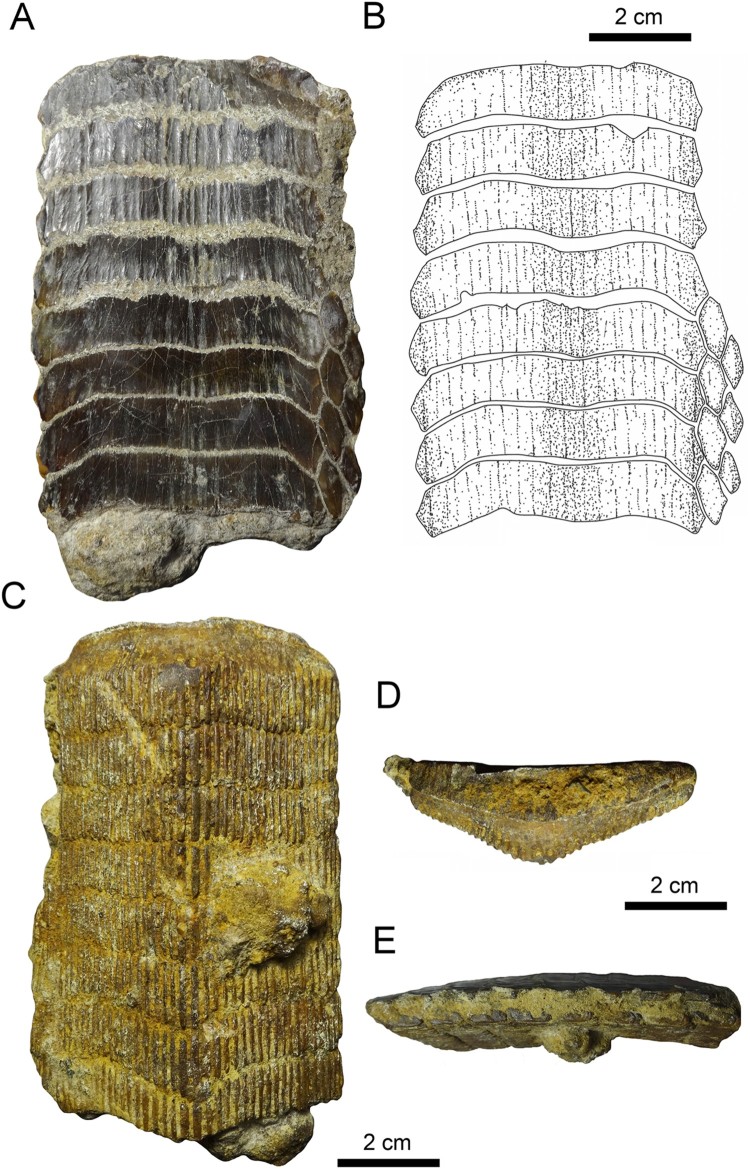
Fossil specimen RK/17-101 from the Bahia Inglesa Formation (middle Miocene–early Pleistocene), Caldera, Chile. **A**, **C**–**E**, lower dental plate; **B**, reconstruction. **A**, **B**, occlusal, **C**, basal, **D**, lingual, and **E**, lateral views.

**FIGURE 6.**
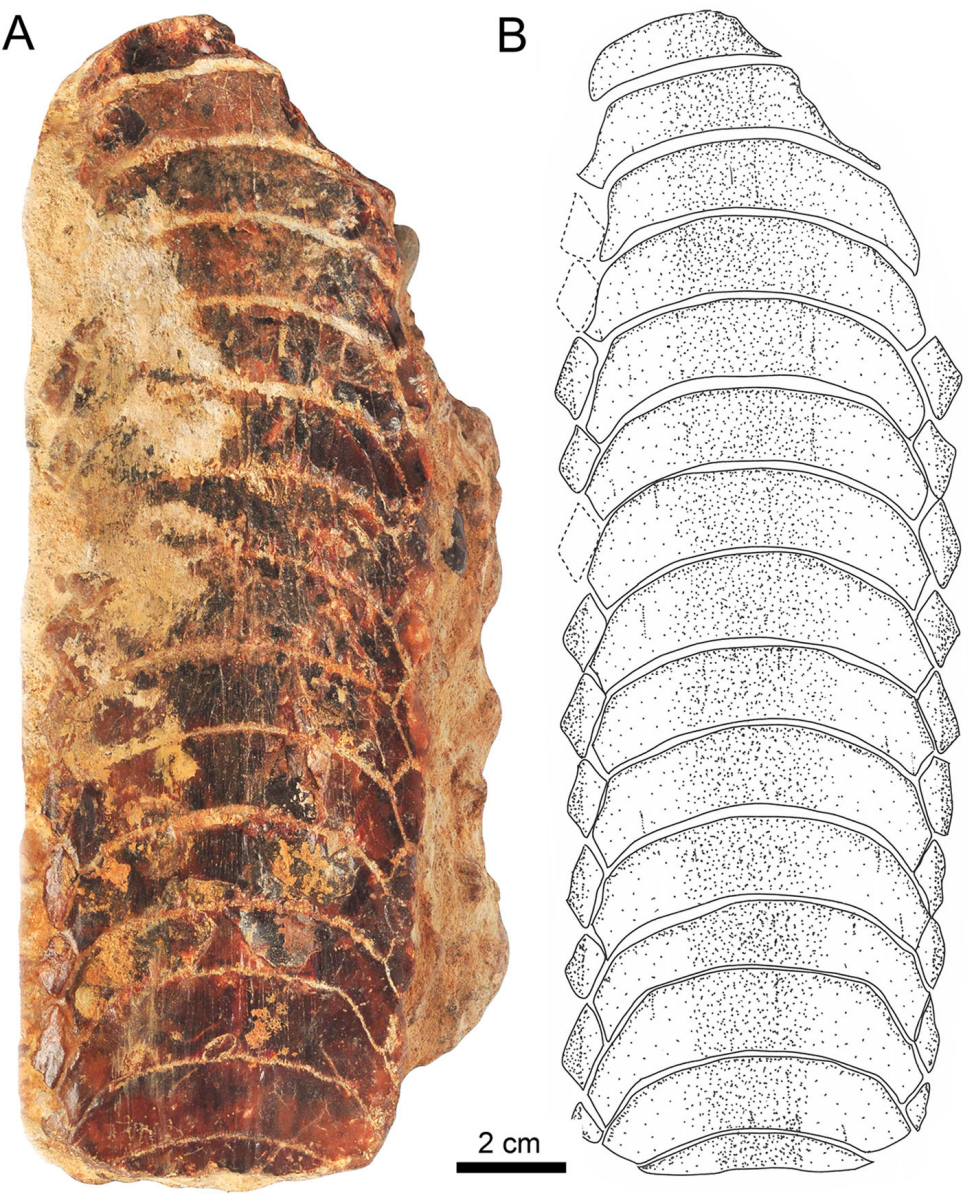
Fossil specimen MPC-34 from the Bahia Inglesa Formation (middle Miocene–early Pleistocene), Mina Fosforita, Caldera, Chile. **A**, lower dental plate and **B**, reconstruction in occlusal view.

**FIGURE 7.**
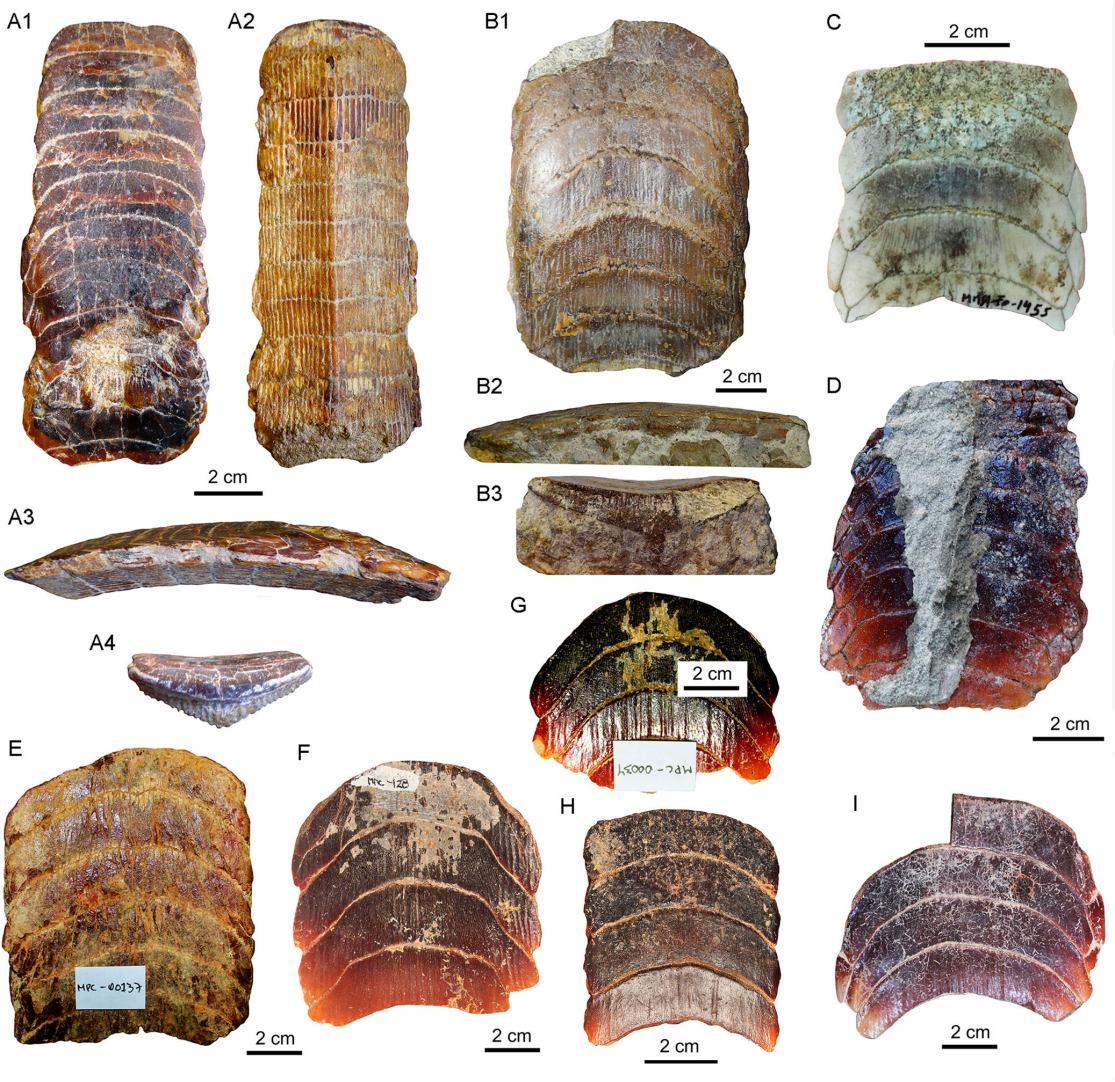
Dental plates from the Temperate Pacific coast of South America. **A1**–**A4**, lower dental plate, RK/17-102, from the Pisco Formation (late Miocene) in **A1**, occlusal, **A2**, basal, **A3**, lateral, and **A4**, lingual views. **B1**–**B3**, lower dental plate, RK/17-99, from the Bahia Inglesa Formation (middle Miocene–early Pleistocene), Caldera, Chile, in **B1**, occlusal, **B2**, lateral, and **B3**, labial views. **C**–**I**, lower dental plates in occlusal view: **C**, MUSA-1455 from the Bahia Inglesa Formation (middle Miocene–early Pleistocene), Bahia Salado, Caldera region, Chile; **D**, SCBUCN-6007 from the Bahia Inglesa Formation (middle Miocene–early Pleistocene), Bahia Salado, Caldera region, Chile; **E**, MPC-137 from the Lacui Formation (early Miocene), Lemuy, Chiloe Island, Chile; **F**, MPC-34, **G**, MPC-128, and **H**, MPC-210 from the Bahia Inglesa Formation (middle Miocene–early Pleistocene), Mina Fosforita, Caldera region, Chile; **I**, SCBUCN-6006 from the Coquimbo Formation (middle Miocene–Pliocene), Quebrada Honda, Coquimbo, Chile.

**Material**—One upper dental plate (MUSA-567) and 12 lower dental plates (MUSA-1455, RK/17-99, RK/17-100, RK/17-101, RK/17-102, SCBUCN-6007, SCBUCN-6006, MPC-34, MPC-36, MPC-128, MPC-137, MPC-210).

**Localities and Age**—RK/17-102 (Fig.7A) comes from the Pisco Formation (late Miocene), Sacaco, Arequipa, Peru; MUSA-567 (Fig. 3) and MUSA-1455 (Fig. 7C) are from the Bahia Inglesa Formation (middle Miocene–early Pleistocene), Bahia Salado, Caldera region, Chile; RK/17-101 (Fig. 5), RK/17-100 (Fig. 4), and RK/17-99 (Fig. 7B) are from the Bahia Inglesa Formation (middle Miocene–early Pleistocene), Caldera, Caldera region, Chile; SCBUCN-6007 (Fig. 7D) was recovered from the Bahia Inglesa Formation (middle Miocene–early Pleistocene) at Punta Cachos in the Caldera region, Chile; MPC-34 (Fig. 7G), MPC-36 (Fig. 6), MPC-128 (Fig. 7F), and MPC-210 (Fig. 7H) also are from the Bahia Inglesa Formation (middle Miocene–early Pleistocene), Mina Fosforita, Caldera region, Chile; SCBUCN-6006 (Fig. 7I) is from the Coquimbo Formation (middle Miocene–Pliocene), Quebrada Honda, Coquimbo, Chile; MPC-137 (Fig. 7E) is from the Lacui Formation (early Miocene), Lemuy, Chiloe Island, Chile.

## DESCRIPTION

**Upper Plate**—Specimen MUSA-567 (Fig. 3), which is an upper dental plate, measures 7.6 cm in labiolingual length and 8.3 cm in total width. It displays eight preserved symphyseal teeth, which are hexagonal in shape, transversally extended, and have only very faintly lingually curved lateral extremities (Fig. 3A, B). The lateral edges of the symphyseal teeth are more or less straight in occlusal view. The width of the median teeth is five to seven times greater than their labiolingual length. The surfaces of the symphyseal tooth crowns are devoid of any ornamentation and are completely smooth. The root of the symphyseal teeth is polyaulacorhizous, with more than 45 enlarged, labiolingually directed laminae separating the nutritive groves (Fig. 3C).

Only a single row of lateral teeth is preserved on each side of the symphyseal row. The lateral teeth are lozenge-shaped and labiolingually longer than wide (Fig. 3A–C). The lateral and lingual margins are equal sized. The root is well preserved and displays the characteristic polyaulacorhizous vascularization pattern (Fig. 3C).

**Lower Plates**—Specimen RK/17-100 (Fig. 4) is a lower dental plate, which measures 11 cm in total labiolingual length and 11.3 cm in total width. The symphyseal teeth are transversally elongated, with distinctly lingually curved extremities (Fig. 4A, B). The specimen displays six preserved hexagonal symphyseal teeth, with the anterior-most tooth being damaged labially (Fig. 4A, B). The lateral edges of the symphyseal teeth in the lower dental plate (Fig. 4A, B) are more obtuse and less markedly acute than in symphyseal teeth of the upper dental plate (Fig. 3A, B). The width of the lower symphyseal teeth is six to seven times wider than long. The coronal surface of the symphyseal teeth displays labiolingually directed grooves resulting from deterioration. In lateral view, the tooth root is higher than the crown (Fig. 4C), similar to the condition seen in specimen RK/17-99 (Fig. 7B2)

One row of lateral teeth is preserved on each side of the symphyseal row, with a rather straight lateral edge. The lateral teeth are lozenge-shaped and labiolingually extended instead of being trapezoidal in occlusal view (Fig. 4A, B). The margins are commonly irregularly shaped. One row of lateral teeth is preserved, comprising teeth with a straight distal edge.

The root displays the polyaulacorhizous vascularization type, with narrow laminae and deep nutritive grooves (Fig. 4D). The root grooves are filled with matrix and thus cannot be properly observed. The lateral view shows a slightly convex occlusal surface (Fig. 4C). The roots of the symphyseal teeth are rather massive medially but decrease in thickness laterally. The morphology of the preserved lateral teeth and the thick, ‘V’-shaped root of the symphyseal teeth (Fig. 4D) are similar to the condition seen in RK/17-101 (Fig. 5D). Specimen RK/17-101 (Fig. 5), which is a lower dental plate, measures 9.8 cm in labiolingual length and 7.3 cm in total width. The specimen displays eight preserved, hexagonal symphyseal teeth, which are transversally elongated and slightly ‘M’-shaped in occlusal view (Fig. 5A, B). The lateral extremities of the symphyseal teeth are slightly lingually curved (Fig. 5A, B). The symphyseal teeth have unequal-sized labio- and linguolateral margins in anterior as well as in posterior teeth. The curvature of the symphyseal teeth is less pronounced (Fig. 5A, B) than in specimen RK/17-100 (Fig. 4A, B). They are about five to six times wider than long and slightly unequal in size. In occlusal view, the crown is ornamented with a well-marked vertical depression and ridges (Fig. 5A, B).

The lateral teeth are labiolingually extended, with obliquely directed long axes (Fig. 5A, B). Only four teeth are preserved in the first right lateral row, whereas three teeth are present in the second lateral row. Teeth in the second lateral row are smaller than teeth in the first lateral row. Teeth of the first row are hexagonal, whereas teeth of the second row are lozenge-shaped (Fig. 5A, B).

The root is heavily abraded (Fig. 5C), but remnants of the distinct, labiolingually directed root grooves are preserved, displaying the characteristic polyaulacorhizous root vascularization pattern (Fig. 5D) as in specimen RK/17-100 (Fig. 4D). The root grooves are, unfortunately, filled with matrix, preventing examination of nutritive foramina (Fig. 5C). In basal view, 38 fine, enlarged laminae can be distinguished in each symphyseal tooth (Fig. 5C). In lingual view, the root is higher in the middle part, but thinning toward the lateral margins gives the root of the symphyseal teeth a slightly ‘V’-shaped pattern (Fig. 5D), similar to the condition seen in specimen RK/17-100 (Fig. 4D). In lateral view, the occlusal surface of the dental plate and the basal root plane are only very faintly convex (Fig. 5E).

Eight lower dental plates share similar dental characters with those described above (Figs. 6, 7). The symphyseal teeth are transversally extended, and the lateral extremities are distinctly lingually curved. Curvature of symphyseal teeth varies to some extent from very strong, as in MPC-36 (Fig. 6) and MUSA-1455 (Fig. 7C), to only slightly curved, as in SCBUCN-6007 (Fig. 7D). The lateral extremities of symphyseal teeth in RK/17-99, RK/17-101, RK/17-102, SCBUCN-6007, SCBUCN-6006, MPC-34, MPC-36, MPC-128, MPC-137, MPC-210, and MUSA-1455, which all represent lower dental plates, are more lingually curved than in specimens MUSA-567 (upper dental plate) and RK/17-100 (lower dental plate; Figs. 3A, B, 4A, B). Specimens MUSA-1455 (Fig.7C) and SCBUCN-6007 (Fig.7D) display hexagonal symphyseal teeth, whereas these teeth are less hexagonal in all other dental plates (Fig. 7A1, B1). The symphyseal teeth are four to five times wider than long. The lateral portions are divided into a labial and a lingual part joining in a more or less acute edge, in which the labiolateral margin is significantly longer than the linguolateral one, forming a blunt angle of approximately 140° as in specimen RK/17-102 (Fig. 7A1). The largest lower dental plate, MPC-36 (Fig. 6A, B), displays 15 preserved symphyseal teeth and measures 21 cm in total labiolingual length and 7 cm in total width.

In lateral view, all lower dental plates are slightly convex, as, e.g., in specimens RK/17-99 (Fig. 7B2) and RK/17-102 (Fig. 7A3). The occlusal surfaces of the symphyseal and lateral teeth all are smooth.

The lateral teeth are lozenge-shaped, labiolingually extended (Fig. 7A1, B1, and C), and arranged in an alternating pattern in the notches formed by two adjacent symphyseal teeth (Fig. 6A3). In SCBUCN-6007 (Fig. 7D), the long axes of the lateral teeth are obliquely oriented, whereas they are labiolingually oriented in specimens MPC-36 (Fig. 6A, B) and RK/17-102 (Fig. 7A1). In specimens MPC-34, MPC-128, MPC-137, MPC-210 and SCBUCN-6006 (Fig. 7E–I), the lateral teeth are not preserved, but it is still possible to detect their original arrangement through the characteristic lateral margins of the symphyseal teeth. The lateral teeth in specimen SCBUCN-6007 (Fig. 7D) are hexagonal, whereas all other specimens have rectangular lateral teeth as in specimen MUSA-1455 (Fig. 7C). In most of the tooth plates, only one row of lateral teeth is present (Fig. 7A1, B1, C). We assume that other more lateral teeth originally were present, but that they were lost during fossilization, recovery, or preparation (e.g., in specimen SCBUCN-6007; Fig. 7D).

The root displays the characteristic polyaulacorhizous vascularization pattern, with deep nutritive grooves separated by numerous quite thin laminae (Fig. 7B3). In lingual view, the root is very thick medially but thinning laterally (e.g., Fig. 7A4). This character is commonly observed in all *Aetomylaeus* specimens. In basal view, 32 elongated laminae are present in each symphyseal tooth (Fig. 7A2). The lateral view shows a slightly curved occlusal surface of the dental plates (Fig. 7A3, B2).

## DISCUSSION AND CONCLUSIONS

**FIGURE 8.**
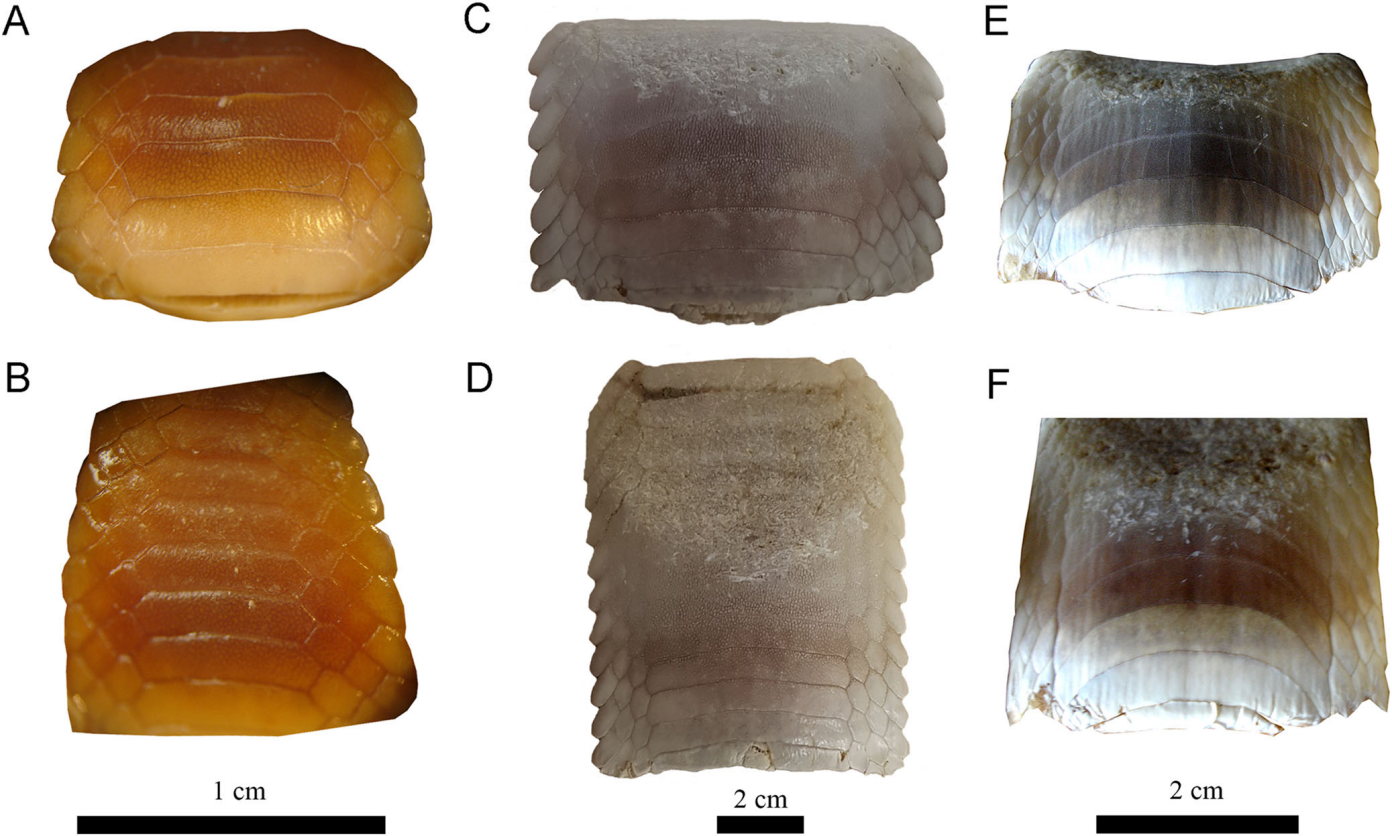
Specimens of extant species of *Aetomylaeus*. **A**, **B**, *Aetomylaeus milvus*, NBC 7465, male from off Djakarta, Indonesia; **C**, **D**, *Aetomylaeus caeruleofasciatus*, CSIRO H 8109-01, adult male from Gulf of Papua, New Guinea; **E**, **F**, *Aetomylaeus bovinus*, ERB DUR02359.88, male from Durban, South Africa. **A**, **C**, **E**, upper plate; **B**, **D**, **F**, lower plate.

**Variations of Dental Characters**—Last et al. (2016) stated that the number of tooth rows in *Aetomylaeus* dental plates is commonly seven (e.g., *A. milvus*; Fig. 8A, B). However, eight tooth rows are present in the extant blue-banded eagle ray (*A. caeruleofasciatus*) from Papua New Guinea (Fig. 8C, D) and in the duckbill eagle ray (*A. bovinus*) from South Africa (Fig. 8E, F). Differences in the number of rows have been previously discussed and attributed to ontogenetic variation in four extant species (e.g., Hovestadt and Hovestadt-Euler, 2013): *A. maculatus*, *A. nichofii*, *A. milvus*, and *A. vespertilio.* Fossil specimens with roots still partly embedded in sedimentary matrix preserve at least one lateral tooth row adjacent the symphyseal row, as in MPC-36 (Fig. 6). On the other hand, lateral teeth generally are not preserved and the lateral margins are more eroded in dental plates that were completely prepared (matrix infill no longer present), as in, e.g., MPC-128 (Fig. 7F). This indicates that teeth of the lateral rows are quite loosely attached to the symphyseal tooth row and easily become separated after death, during burial or even preparation. According to Enault et al. (2013) and Underwood et al. (2017), the reduction of lateral tooth rows might be related to the origin of planktivory in batoids. Consequently, the number of tooth rows seemingly is not useful for species identification in *Aetomylaeus*. Moreover, the considerable intraspecific variation of the examined extant and fossil material described above might be related to ontogenetic heterodonty patterns, but not exclusively. We consequently refrain from assigning the material described here to any species because the variability of tooth row numbers and the morphological variation within tooth rows in extant and extinct *Aetomylaeus* species still is not well established and because the material that forms the focus of this study might not be completely preserved. We also suggest that researchers should be careful in naming fossil species of *Aetomylaeus*. Studies focusing on the revision of dental characters of this genus would help to understand better these variations.

**Stratigraphic and Paleogeographic Distribution**—The stratigraphic distribution of *Aetomylaeus* is still ambiguous due to the fact that some species erroneously were assigned to either *Myliobatis* or *Aetobatus* without detailed descriptions (Cappetta, 2012; Hovestadt and Hovestadt-Euler, 2013). However, the oldest records that are currently considered to represent *Aetomylaeus* come from Eocene strata (Cappetta, 2012). Cicimurri and Ebersole (2015) additionally reported the presence of *Aetomylaeus* in Eocene localities of North America. Remains of *Aetomylaeus* from Chile previously were indicated to occur in the late Miocene of Bahia Inglesa (Suárez et al., 2004; Gutstein et al., 2008). However, these records remain ambiguous because of the lack of any morphological description or figure. Moreover, possible misidentification could be present in specimens from the eastern Pacific; therefore, the material needs to be reexamined in detail.

Today, *Aetomylaeus* is absent from the southeastern Pacific along the Peruvian and Chilean coasts but occurs with *A. asperrimus* in the central Pacific (off Panama and Galapagos islands). Reasons for the absence in Peruvian and Chilean waters might be related to climatic preferences of this group. Cione et al. (2007) and Villafaña and Rivadeneira (2018) reported changes in the paleobiogeographic distribution of chondrichthyans from the TPSA, although they were less affected than other marine vertebrates (i.e., mammals) by global extinctions since the Neogene (Villafaña and Rivadeneira, 2014). For tropical America, Carrillo-Briceño et al. (2018) revealed an extirpation/extinction process affecting at least 24 genera of sharks and five rays in the Americas during Neogene times, likely as a direct consequence of environmental changes post closure of the Central America Seaway. Thermal tolerance limitation is one of the most common explanations for regional and global extinctions of chondrichthyans (Cione et al., 2007; Villafaña and Rivadeneira, 2014, 2018; Pimiento et al., 2016; Carrillo-Briceño et al., 2018). Thus, the tropical preference of the extant rough eagle ray *A. asperrimus* in the central Pacific most likely is the reason for its absence in the southeastern Pacific today. During the Neogene, tropical conditions still prevailed in the TPSA, supporting the presence of *Aetomylaeus* in this region (Villafaña and Rivadeneira, 2018). Warm water preferences also were suggested for the extinct species †*Aetomylaeus cubensis* from Central America (Aguilera et al., 2017). Additionally, extant *Aetomylaeus* species commonly inhabit soft-bottom environments, reaching a maximum depth of 150  m (Last et al., 2016). Recently, Villafaña and Rivadeneira (2018) observed that genera that inhabit deeper waters have a higher probability of contracting their southern latitudinal range in the TPSA. The onset of the oxygen minimum zone during the Neogene in Chile (Martinez-Pardo, 1990) might have caused a restriction in their vertical distribution. In addition, the restricted distribution range of the extant *A. asperrimus* might be the result of climatic changes and its subsequent extirpation from the Chilean and Peruvian coast since the Neogene. Therefore, the cooling effect during the Neogene (Zachos et al., 2001) seemingly might have been the principal reason for the regional extinction of *Aetomylaeus* along the southeastern Pacific coast.
